# The *X* Ray in the Law Courts

**Published:** 1900-05-26

**Authors:** 


### THE *X* RAY IN THE LAW COURTS.

When the *x* rays and their powers were first discovered very high expectations were formed as to the enlightenment which we should obtain in regard to the nature of many internal derangements caused by accident or by disease, and, in some ways, early expectations have been more than realised. Experience, however, has shown that the problem is not so simple and straightforward a one as some people had imagined, and that many precautions, at first unthought of, have to be taken in properly interpreting the results obtained by skiagraphy. Among experts, of course, all this soon came to be thoroughly understood; but the public had been so taken with the new invention, and the common man is so convinced that "seeing is believing," that considerable risk has been and still is run by medical men in consequence of the readiness of juries to be swayed in medico-legal cases by the "evidence" of their senses, as perceived in that most fallacious thing, a skiagraph. It is well, then, to direct attention to the report recently presented to the American Surgical Association by Dr. William White, Professor of Clinical surgery in the University of Pennsylvania, on the medico-legal relations of the *x* rays. In this document it is reported that there have already been cases of improper use of the skiagraph in court; that there is a real danger of people being led by the undoubted advantages of skiagraphy to exalt it beyond its present merits; that important mistakes have been made in reference to fractures, foreign bodies, and renal calculi; that suits for damages on account of $x$ ray burns are now occurring; and that to counterbalance the extremists many members of the profession have called attention to the fallacies of skiagraphs and the danger of their use in medico-legal cases. Among other things mentioned is that the employment of the $x$ ray in cases of fracture is not of sufficiently definite advantage to justify the teaching that it should be used in every case, although when doubt arises it, as well as every other means, should, of course, be employed. What we think is especially important is the statement that in competent hands plates may be made which will fail to reveal the presence of existing fracture or will appear to show a fracture which does not exist. Again, it is shown that in questions of deformity skiagraphs alone, without expert surgical interpretation, are generally useless and frequently misleading. "The appearance of deformity may be produced in any normal bone, and existing deformity may be grossly exaggerated." As to callus after fracture, it is stated that fibrous union cannot be distinguished from union by callus in which lime salts have not yet been deposited, and that the testimony of the $x$ rays in such cases is especially fallible. Many other points are raised in this interesting report, but enough has been said to show that great as are the advantages of using the $x$ ray as an adjunct to other sources of information by the summation of which a diagnosis may be arrived at, nothing can be more dangerous than the popular belief that vision cannot mislead and that on the strength of a skiagraph produced in court a charge of malpractice can properly be established.

